# Association between body image perception with demographic characteristics of physically active individuals during COVID-19 lockdown in Saudi Arabia

**DOI:** 10.3389/fspor.2022.985476

**Published:** 2022-10-28

**Authors:** Mezna A. AlMarzooqi

**Affiliations:** Department of Community Health Sciences, College of Applied Medical Sciences, King Saud University, Riyadh, Saudi Arabia

**Keywords:** body image, body image dissatisfaction, COVID-19, physically active, Saudi Arabia

## Abstract

**Objective:**

This study aimed to determine the relationship between body image perception and demographic factors among physically active (men and women) during the COVID-19 lockdown in Saudi Arabia.

**Methods:**

A descriptive cross-sectional survey was employed among physically active individuals in Saudi Arabia between June and July 2020. Eligible participants completed a 19-item self-administered questionnaire that covered three areas: demographic questions, reasons for physical activity, and role or perceptions of body image during the COVID-19 pandemic quarantine.

**Results:**

A total of 323 physically active individuals participated in this study. The majority of the participants were female (*N*=217, 72.7%), were married (66.6%), and living in the Middle region of Saudi Arabia (*N*= 268, 83%). The analysis shows that majority of the participants were dissatisfied with their current body shape. The analysis also showed a significant association between participants' educational attainment and BMI and body dissatisfaction (*p*= 0.001). The strongest predictor was BMI level, recording an odds ratio (OR) of 5.99 (CI: 2.15 – 10.54, *p*=0.001) in obese and an OR of 4.55 (CI: 1.31 – 9.35, *p*=0.001) in overweight, indicating that compared with normal weight, obese and overweight participants were more likely to be dissatisfied by five and four times, respectively.

**Conclusion:**

This study indicates that physically active individuals are greatly influenced by the confinement period. Programs that promote physical activity in their house or during lockdown may help to encourage, lessen their anxiety, and maintain their health. This may also decrease the anxiety of individuals, particularly those active ones.

## Introduction

Saudi Arabia reported the first coronavirus disease 2019 (COVID-19) case on 2 March 2020. The rapid increase of COVID-19 cases and deaths has caused a global public health concern. Different countries implemented nonpharmaceutical interventions (NPIs) to mitigate the spread of COVID-19. The Ministry of Health implemented NPIs such as suspension of all classes, 14 days of isolation for travelers, mask-wearing, physical distancing, the shutdown of nonessential businesses (e.g., coffee shops, sports clubs, schools, and fitness gyms), and a nationwide curfew between 2 p.m. and 6 a.m. (1). In addition, the Islamic pilgrimage called “Umrah,” performed by thousands of Muslims in Mecca, was also suspended to contain the COVID-19 outbreak. Due to the unprecedented measures placed on people's movements may significantly influence their habits and behaviors and consequently increase the risk of anxiety and emotional distress (2).

Several studies have expressed concern about the potential health risk for NPIs, particularly lockdown measures that increase sedentary behaviors and irregular eating patterns in the general population (3–6). In addition, collective anxiety about weight gain during the lockdown and stigmatizing media messages about the dangers of higher body weight might contribute to increased body shame and levels of disordered eating (2). A global study shows that home confinement had a negative effect on the intensity of physical activity of individuals and increased the number of sitting time (7). Other studies also revealed the negative effect of home confinement on the emotional wellbeing of individuals (8, 9).

Physical inactivity increases the risk of many chronic diseases, such as hypertension, coronary heart disease, stroke, diabetes, depression, and risk of falls (10). Previous studies have shown that proper or regular physical activity helps maintain a healthy weight, reduces the risk of developing obesity, and strengthens the immune system (10, 11). Considering the health benefits of regular physical activity, the WHO recommends that individuals, mainly adults, undertake 150–300 min of moderate-intensity, or 75–150 min of vigorous-intensity physical activity, or some equivalent combination of moderate-intensity and vigorous-intensity aerobic physical activity per week (12).

To individuals who are physically active and involved in sports disciplines and where appearance, including body shape and mass, is essential, the effects of lockdown measures can increase the level of anxiety from undertaking physical activity. Furthermore, individuals who exercise regularly can be expected to differ in the impact of NPIs because it tends to be more concerned and dissatisfied with their appearance compared with those who are physically inactive. Body image dissatisfaction is defined as negative thoughts, feelings, and perceptions about one's body (13). Body image is a multidimensional concept of individual perception, affection, and behaviors (e.g., satisfaction or dissatisfaction with body image and evaluation of body size) (14, 15). Body image involves different aspects, such as cognitive, emotional, social, and cultural, in addition to dissatisfaction with own body (14). A popular belief about body image is that social media and society with lean anthropometric profiles are beauty standards (16, 17). Moreover, physical activity is regarded as a critical component of a healthy lifestyle and disease prevention (18). A previous study has shown that a negative body image may work as both a motivation and a barrier to exercise participation (19).

Several studies revealed that body weight concerns and body dissatisfaction are prevalent in Arab countries, particularly among women. A survey conducted in five Arab countries (Syria, Bahrain, Jordan, Oman, and Egypt) shows that 33% of Arab women were dissatisfied with their body weight (20). In Saudi Arabia, 21.4% of men and 33.5% of women reported body image dissatisfaction (21). Another study among Saudi females attending fitness centers revealed that 87% were dissatisfied with their body image, including those of normal weight (22). Such findings may call the attention that the COVID-19 and NPIs will have negative implications for those individuals, particularly those who are physically active.

Accordingly, it is necessary to identify the perceived changes to their body image behaviors during this period considering the effects of the COVID-19 lockdown. We hypothesized that social isolation, lockdown, and quarantine in Saudi Arabia may have adversely impacted the perception of body image of physically active individuals. Thus, we aimed to assess the body dissatisfaction of physically active (men and women) during the COVID-19 lockdown in Saudi Arabia and determined the possible associated factors that can serve as a baseline for future policies and health programs.

## Materials and methods

### Design and participants

This descriptive cross-sectional study employed a total of 323 physically active individuals who were living in Saudi Arabia during the COVID-19 pandemic. The participants' inclusion criteria were as follows: aged 18 years and above, Saudi nationals, physically active individuals exercising equal to or more than 150–300 min of moderate activity for 1 week, and member of a sports club. The participants were also asked to describe themselves as somehow active, amateur, and athletes for the classification of physical activity. A convenience sampling method was used due to the pandemic situation. A 5% margin of error, a confidence level of 95%, and a significance value of 0.05 were used as statistical parameters. Ethical approval for this study was approved by the King Saud University Ethics Research Committee. All participants provided informed consent prior to participating in this study.

### Instrument

An instrument was developed which covered the following three areas: demographic questions, role and perception of body image, and reasons for physical activity during the COVID-19 quarantine. The demographic section includes age, sex, marital status, educational level, and body mass index (BMI). The Stunkard Figure Rating Scale (FRS) images were used to assess the perception of the body image of the participants. This questionnaire consists of two diagrams in which the first diagram ranks their actual body image based on the nine silhouette figures, while the second diagram ranks their desires to look like from the same nine figure images. A corresponding rating score of each figure is from 1 to 9, with nine representing the most obese figure and one the thinnest (23). The discrepancy between perceived and desired body image scores was calculated. Body dissatisfaction occurred when the desired silhouette was smaller than the self-evaluated and dissatisfaction with slimness when the desired shape was larger than the self-evaluated (23).

The International Physical Activity Questionnaire (IPAQ) was used to assess participants' physical activity levels. The participants were asked how often (the number of days per week) and for how long (the average time in minutes) they had been active at light, moderate, and vigorous intensities during the last 7 days. The intensities of physical activity were assigned to an average Metabolic Equivalent Time (MET) to yield MET-minutes (MET-min) per week. We based the overall MET value on the average MET value for each intensity in the MET compendium (24, 25). Light activity was 3.3 MET, moderate 4.0 MET, and vigorous 8.0 MET. All scored data were according to the IPAQ scoring protocol, version 2.0. All participants in one or more intensity levels had reported days (frequency) and time (duration) of physical activity or vice versa and were included in the analysis by summing up the frequency and duration of activity. A draft questionnaire was piloted and revised to a final survey of 19 items related to electronic survey response and demographic variables, which took approximately 15 min to complete. All the participants' responses were recorded *via* the platform of the study survey and downloaded by a trained researcher.

### Data gathering procedure

All participants were recruited online through a sports club group. All eligible participants received an invitation, including a link to an online survey. The survey questionnaire includes participants' consent information, which explains the rationale for the study, and all participants' information is confidential and anonymous. All participants entered and completed the survey between June and July 2020.

### Statistical analysis

Statistical analysis was performed using SPSS (version 23.0). Microsoft Excel was used for data entry, editing, and sorting. Continuous data were presented as mean and standard deviation (SD), and categorical data as frequency and percentage. The classifications of physical activity were categorized into three based on the guidelines and recommendations of the International Physical Activity Questionnaire Research Committee (IPAQ Research Committee, 2005). The proposed levels of physical activity are [i] inactive (low), [ii] minimally active (moderate), and [iii] health-enhancing physical activity, or a high active category (IPAQ Research Committee, 2005). Furthermore, the level of physical activity was dichotomized into moderate activity, which has >1,680 MET-min/week, and vigorous activity, whose MET-min/week was < 1,680 (24–26). The association between body image and physical activity was examined using the chi-square test analysis. To assess the predictors associated with body dissatisfaction, we performed a multiple logistic regression model, and the odds ratios (ORs) and 95% confidence intervals (CIs) were obtained. The dependent variable was body image, and the reference category was “Satisfied”. Statistical significance was set at *p* < 0.05.

## Results

The composition of the study sample with age category shows that 29.1% were aged 18–24, 24.5% were aged 25–29 years, 17.3% were aged 30–34 years, 16.4% (26–30), and 12.7% were aged 40 years and above (Table 1). The majority of the participants were female (*N* = 217, 72.7%), were married (66.6%), and living in the Middle region of Saudi Arabia (*N* = 268, 83%). Sixty percent of the participants were of university education level, where a small sample had a high school/diploma (18.6%) or < high school (0.6%). Most of the respondents were amateurs (*N* = 220, 68.1%), and 26% (*N* = 85) were athletes in the club or the school. More than half of the participants had normal BMI levels (*N* = 204, 63%), 27.9% (*N* = 90) were overweight, and 9% of the participants were obese (*N* = 29). Notably, 31% of the participants had higher energy expenditure in MET-min/week of vigorous based on the 1,680 MET-min/week cutoff. The majority of the participants were dissatisfied with their current body shape or desired a smaller shape (*N* = 276, 85.5).

**Table 1:** Demographic characteristics of the participants.

**Variable**	***N* = 323**	**%**
**Age**		
18–24	94	29.1
25–29	79	24.5
30–34	56	17.3
35–39	53	16.4
40 and above	41	12.7
**Gender**		
Male	66	27.3
Female	217	72.7
**Marital status**		
Single	108	33.4
Married	215	66.6
**Educational level**		
< high school	2	0.6
High school/diploma	60	18.6
Bachelor's degree	196	60.7
High education degree	65	20.1
**Region**		
Middle region	268	83
Eastern and Western region	47	14.5
Northern and Southern Western	8	2.5
**BMI**		
Normal	204	63.1
Overweight	90	27.9
Obese	29	9
**Classification of physical activity**		
I am not always active	18	5.6
Amateur	220	68.1
Athletes in the club or school	85	26.3
**Level of physical activity**		
METs-min/week from moderate activity < 1,680	222	68.1
METs-min/week from vigorous activity > 1,680 METs-min/week	103	31.9
**Body dissatisfaction**		
Satisfied	47	14.5
Dissatisfied	276	85.5

Table 2 displays the demographic characteristics of physically active individuals who were satisfied and dissatisfied with their body shape. The analysis shows that majority of the participants were dissatisfied and desired a smaller shape. The chi-square test analysis also indicated a significant association of participants' educational attainment and BMI with body dissatisfaction (*p* = 0.001). The proportion of physically active individuals with body dissatisfaction across educational attainment increased significantly (high school/diploma−77.4%, Bachelor's degree−85.7%, and Postgraduate or High education degree−92.3%, *p* < 0.05). Body dissatisfaction was also significantly associated with participants' BMI in which the majority of the participants who were overweight (*N* = 65, 72%) and obese (*N* = 23, 79.3%) were dissatisfied with their actual BMI (*p* = 0.001). The analysis also shows that 92.2% who had normal BMI were dissatisfied or desired a smaller shape than their actual BMI (*p* = 0.001).

**Table 2:** Association of body image dissatisfaction with demographic characteristics of the participants.

		**Body Image**		
**Variable**	***N* = 323**	**Satisfied (*N* = 47)**	**Dissatisfied (*N* = 276)**	***P*-value**
**Age**				0.568
18–29	173 (53.6)	23 (13.3)	150 (86.7)	
30–39	109 (33.7)	19 (82.6)	19 (17.4)	
40 and above	41 (12.7)	5 (87.8)	36 (12.2)	
**Gender**				0.175
Male	66 (27.3)	19 (17.6)	89 (82.4)	
Female	217 (72.7)	28 (13)	187 (87)	
**Marital status**				0.211
Single	108 (33.4)	18 (16.7)	90 (83.3)	
Married	215 (66.6)	29 (13.5)	186 (86.5)	
**Educational level**				0.050
High school/diploma	62 (19.2)	14 (22.6)	48 (77.4)	
Bachelor's degree	196 (60.7)	28 (14.3)	168 (85.7)	
High education degree	65 (20.1)	5 (7.7)	60 (92.3)	
**Region**				0.091
Middle region	268 (83)	44 (16.4)	224 (83.6)	
Eastern and Western region	47 (14.5)	2 (4.3)	45 (95.7)	
Northern and Southern Western	8 (2.5)	1 (12.5)	7 (87.5)	
**BMI**				0.001
Normal	204 (63.2)	16 (7.8)	188 (92.2)	
Overweight	90 (27.9)	25 (27.8)	65 (72.2)	
Obese	29 (9)	6 (20.7)	23 (79.3)	
**Classification of physical activity**				0.094
I am not always active	18 (5.6)	4 (22.2)	14 (77.8)	
Amateur	220 (68.1)	32 (14.5)	182 (85.5)	
Athletes in the club or school	85 (26.3)	11 (12.9)	74 (87.1)	
**Level of physical activity**				0.311
METs-min/week from moderate activity < 1,680	222 (68.1)	34 (15.5)	186 (84.5)	
METs-min/week from vigorous activity > 1,680 METs-min/week	103 (31.9)	13 (12.6)	90 (87.4)	

Chi-square tests for the differences in proportions; significance level at < 0.05.

Multiple logistic regression was performed to assess the predictors of body dissatisfaction of physically active individuals. The model contained eight independent variables (age, gender, marital status, educational level, region, BMI level, classification of physical activity, and level of physical activity). As shown in Table 3, only one variable emerged as a significant predictor of body dissatisfaction of physically active individuals. The strongest predictor was BMI level, recording an OR of 5.99 (CI: 2.15–10.54, *p* = 0.001) in obese and an OR of 4.55 (CI: 1.3– 9.35, *p* = 0.001) in overweight, indicating that compared with normal weight, obese and overweight participants were more likely to be dissatisfied by five and four times, respectively.

**Table 3:** Predictors associated with body dissatisfaction among physically active individuals.

	**OR (95 % CI)**	***P*-value**
**Variable**	*N* = 323	
Age		0.820
18–29	1	
30–39	1.11 (0.45–2.74)	
40 and above	0.36 (0.08–1.49)	
**Gender**		0.649
Male	1	
Female	0.84 (0.08–1.49)	
**Marital status**		
Single	1	
Married	1.36 (0.53–3.48)	
**Educational level**		0.099
High school/diploma	1	
Bachelor's degree	0.57 (0.25–1.29)	
High education degree	0.26 (0.07–0.90)	
**Region**		
Middle region	1	0.094
Eastern and Western region	0.18 (0.04–0.84)	
Northern and Southern Western	0.94 (0.09–9.30)	
**BMI**		0.001
Normal	1	
Overweight	4.55 (2.22–9.35)	
Obese	5.99 (2.15–10.54)	
**Classification of physical activity**		
I am not always active	1	0.819
Amateur	0.77 (0.21–2.79)	
Athletes in the club or school	0.64 (0.15–2.70)	
**Level of physical activity**		
METs-min/week from moderate activity < 1680	1	0.92
METs-min/week from vigorous activity >1680	0.96 (0.45–2.20)	

CI, confidence interval for odds ratio (OR); dependent variable “body image” and the reference category “Satisfied”; significance level at < 0.05.

## Discussion

This study intended to assess the body dissatisfaction of physically active (men and women) during the COVID-19 lockdown in Saudi Arabia. The analysis shows that majority of the participants were dissatisfied with their body image or desired a smaller shape. The findings were parallel with previous research among Saudi females attending fitness clubs and those adolescents in Brazil (14, 22). The less altered body image dissatisfaction of the participants was possibly driven by the concerns during a lockdown of not engaging in their usual physical activity routine. A previous study concurred that body dissatisfaction is associated with disordered eating (19). Behavior theory suggests that a common emotional response to a pandemic is an exaggerated feeling of fear or anxiety (31, 32). Therefore, these findings may support concerns that the COVID-19 lockdown is a factor in developing these complex health conditions.

Our results also highlight that educational attainment and BMI level were significantly associated with body dissatisfaction. These results confirm that body shape dissatisfaction was higher than those with higher education, as previously stated in other literature (33). With regards to body dissatisfaction between BMI levels, it was noted that the majority of participants with normal BMI levels were dissatisfied or desired a smaller shape than their actual BMI. Similar findings were found among Spanish adults and in the USA (34, 35). Our findings were also parallel to a study in Hong Kong in which the majority of the participants with normal weight status desired a leaner or slimmer body (33). The results are not surprising that a relatively high proportion of individuals with normal BMI levels misperceived their body image since our participants were physically active individuals. This mismatch could also have been perceived by the desire for an ideal body shape influenced by norms, media, and society (33). The findings indicate a signal of a feeling of distress and the development of anxiety among the participants. In addition, misperception of body shape is common, particularly among adolescents and adults (36–38).

Another result worth emphasizing was BMI levels as a predictor associated with the body dissatisfaction of the participants. The analysis indicated that the strongest predictor was obesity, followed by overweight participants in the BMI level. The research reflects the difference between BMI levels and body image perception of physically active individuals during the COVID-19 pandemic. These findings were consistent with previous research in Brazil (14, 17). A previous study suggests that promoting exercise needs to deemphasize weight loss and appearance for positive body image (27, 39). These findings demonstrated the importance of physical activity behavior as a potential mechanism to develop body image positivity. Considering the benefits of physical activity during quarantine and in relation to body image, it is necessary to identify ways and promote exercise during the isolation period. Similarly, physical activity could be recommended to reduce the negative emotional effect during the periods of lockdown and quarantine (2, 28). In addition, digital health solutions such as exergames or the development of virtual coaches can allow easy and accurate interventions as well as recommendations to improve physical activity during the quarantine and pandemic (29).

The findings of this study present some limitations. First, the cross-sectional design of this study limits the causality. Second, the small sample size and the use of convenience sampling may result in bias and may not be able to generalize the entire population. However, the results are comparable with the previously conducted study. In addition, it produced information about body image perception (body dissatisfaction) and its association with the demographic characteristics of physically active individuals during the COVID-19 lockdown in Saudi Arabia.

## Conclusion

This study identified that the majority of the participants were dissatisfied with their current body shape. The analysis also found that the proportion of physically active individuals with body dissatisfaction increased significantly across educational attainment. In addition, the findings indicate a trend of increased body dissatisfaction from normal weight to overweight and then decreased likelihood of obese participants. This study indicates that confinement greatly influences physically active individuals. The findings of this study provide important insights into the effect of COVID-19 and NPIs among physically active individuals during the COVID-19 pandemic. The present results need to be interpreted with caution due to these limitations. The present findings have potential implications that could aid in developing interventions to mitigate the effect of the COVID-19 pandemic, particularly among these individuals. We recommend programs that promote physical activity in their house or during lockdown which may help to encourage, lessen their anxiety, and maintain their health. This may also decrease the feeling of distress of physically active individuals.

## Data availability statement

The data that support the findings of this study are available at the Department of Community Health Sciences, College of Applied Medical Science King Saud University, but restrictions apply to the availability of these data, which were used under license for the current study, and so are not publicly available. Data are however available from the authors upon reasonable request.

## Ethics statement

Institutional Review Board Committee at King Saud University approved the study prior to enrollment in this study. The patients/participants provided their written informed consent to participate in this study.

## Author contributions

MA contributed to data analysis, interpretation of results, drafting, or revising the manuscript, agreed to be accountable for all aspects of the study, and approved the final version of the manuscript.

## Funding

This research project was supported by a grant from the Research Center of the Female Scientific and Medical Colleges, Deanship of Scientific Research, King Saud University.

## Conflict of interest

The author declares that the research was conducted in the absence of any commercial or financial relationships that could be construed as a potential conflict of interest.

## Publisher's note

All claims expressed in this article are solely those of the authors and do not necessarily represent those of their affiliated organizations, or those of the publisher, the editors and the reviewers. Any product that may be evaluated in this article, or claim that may be made by its manufacturer, is not guaranteed or endorsed by the publisher.
